# Prevalence and Correlates of Depression Among School-Going Adolescents in the Urban Schools of Central India: A Cross-Sectional Study

**DOI:** 10.7759/cureus.44088

**Published:** 2023-08-25

**Authors:** Darshan Parida, Pankaj Prasad, Pushpendra Sahu, Subba Krishna Krishna, Ankur Joshi, Deepti Dabar, Sudhir Verma

**Affiliations:** 1 Department of Community Medicine, Index Medical College, Hospital & Research Centre, Indore, IND; 2 Department of Community and Family Medicine, All India Institute of Medical Sciences, Bhopal, IND; 3 Department of Community Medicine, Government Medical College, Satna, IND; 4 Department of Community and Family Medicine, All India Institute of Medical Sciences, Guwahati, IND

**Keywords:** school health, depression, psychological health, mental health, adolescent health

## Abstract

Background: Adolescence is defined as the phase of development that occurs between childhood and adulthood. Presently in India, 243 million populations are staring at the crossroads of transition from childhood to adulthood. Physical, emotional, and social issues unique to this age group make them vulnerable to various mental problems. So, we conducted this study to quantify the current burden of depression in adolescents and its possible causes.

Materials and methods: The present community-based cross-sectional study was conducted in the middle and late adolescent participants aged 14-19 years from 52 sections (clusters) of 9th to 12thclasses comprising a total of 1412 students with a multistage cluster sampling method. In total four sections (clusters), and one participant of class 9th, 10th, 11th, and 12th were chosen from 13 preselected schools. The questionnaire consisted of socio-demographic details, screen time, physical activity, etc., and the DASS-42 scale was used to determine the prevalence of depression.

Results: We found that the prevalence of depression in our study participants was around 39%. It was classified as 16.9%, 16.7%, 5.1%, and 0.5% participants respectively having mild, moderate, severe, and extremely severe depression. Mother’s education was a statistically significant determinant for depression among these adolescents.

Conclusion: The study concludes that the prevalence of depression (including mild, moderate, severe, or very severe) among school-going adolescents is 39%. We hereby recommend that a holistic approach should be followed involving parents and teachers with the help of school counselors to tackle and curb this problem.

## Introduction

The word “Adolescence” is derived from the Latin word “Adolescere” which means “to grow up” [1,2]. There are many ways of defining adolescence, the most common of which is children in the age group of 10 to 19 years [3]. It is the phase of development that occurs between childhood and adulthood, where a child goes through many physical, emotional, and psychological changes along with changes in the child’s social adapting capacity [4]. This phase somehow misses out on the socio-cultural interactions which the child should make while undergoing this biological transition [5]. The WHO classifies adolescence as early adolescence and late adolescence. Early adolescence encompasses the age between 10 and 14 years, whereas late adolescence comprises those belonging to the age between 15 and 19 years [4]. Today one in every five individuals in the world is an adolescent, which constitutes about 1.2 billion population, out of which 350 million reside in SEAR. In India, nearly 243 million populations are on the verge of transition from childhood to adulthood [5].

Adolescent health is the result of various interactions between prenatal and early childhood development as well as the specific biological and social role changes that come with puberty. It is also influenced by the risk and protective factors that affect the uptake of health-related behaviors [6]. They are no longer children, yet they are not adults which makes this group more vulnerable to various emotional and social problems [7]. All these factors along with the performance pressure in schools and among peers make them more vulnerable, ultimately landing up with one or other mental illnesses like depression, anxiety, stress, etc. Depression is the fourth leading cause of illness and disability among adolescents between the age of 15 and 19 years globally [1,8]. The situation in India too is no different as one out of every six adolescents is affected by some or other kind of mental illness of which depression is again the most common [6]. If this problem of depression is not addressed in the early stage, it may extend well into adulthood, thereby impairing their physical, mental, and psychosocial health and will limit their opportunities in the future [1]. There is a wide variety of evidence-based programs in India for the prevention of depression among adolescents, such as Rashtriya Kishor Swasthya Karyakram (RKSK) that emphasizes the need to strengthen the Adolescent Friendly Health Clinics (AFHCs) and Adolescent Reproductive and Sexual Health Programme (ARSH) [9]. However, adolescent mental health in these programs has been included as one of the multi-faceted components and is not being considered as the primary concern creating a huge gap between the mental health needs of adolescents especially depression among school-going adolescents and the current facilities being provided to them. Moreover, the lack of trained personnel having required expertise in this field delays the diagnosis of mental health problems like depression. Thus to quantify the problem of depression in this group, we conducted this study among school-going adolescents with the objectives to determine the prevalence of depression and to understand its associated factors.

## Materials and methods

The study had been carried out in the schools in the catering areas of the Urban Health Centre of All India Institute of Medical Sciences Bhopal (i.e. area catering 1100 quarters and Saibaba Nagar) which was also our sampling frame. This area was chosen for operational feasibility and also for ease of linking with the current health system. All India Institute of Medical Sciences (AIIMS) Bhopal is a tertiary-level health center situated in the Bhopal district of Madhya Pradesh India and has a daily OPD of approximately 4,000 patients. This was a community-based cross-sectional study and was conducted between July 2019 and June 2021. 

A total of 1500 participants were taken for our study. The sample size was calculated by taking the 95% confidence level and a design effect of 4.37 which can out to be 1356. It was rounded off to 1500. It was calculated using the following formula: Sample Size = [Z2 x P(1-P)]/D2. In total, we took 52 sections of the class of the schools for our study. We included all the middle and late adolescent participants (age 14-19 years) attending secondary and higher secondary school and the students fitting strictly into the criteria of age from 14-19 years and class 9th-12th. A multistage cluster sampling method was employed, and a section of a class was deemed to be a cluster of the study. As our sampling frame was secondary and senior secondary schools present in the catering area of the Urban Health Centre of our Institute, firstly a list of these schools was prepared. With the help of a random number generator, the list of schools was arranged randomly and 13 schools were selected randomly from this list which included both Government and private schools. Then, one section of each of the 9th, 10th, 11th, and 12th classes was randomly selected from the 13 selected schools making it a total of 52 sections or clusters. As per the instruction from school authorities, we have given a detailed “participant information sheet” and “consent form” to the parents of study participants. All those who brought back the signed copy of the consent form from their parents were included in the study and detailed information about the study was given before the administration of the study tool. Any students from the selected cluster, who did not consent to the study or were suffering from a prior psychiatric condition, were excluded from the study. The study tool consisted of two parts - Part I consisted of questions based on their socio-demographic details, screen time (which was taken as low when the total time spend using electronic gadgets like mobile, tablets, laptops, t.v. etc. ≤ 2 hrs/ day and high when it was > 2 hrs/ day), physical activity, failure in any class or not, etc. Part II consisted of 42 questions of the Depression Anxiety Stress Scale (DASS-42). DASS-42 is a pre-validated scale in both English and Hindi. It is a free-to-use questionnaire [10]. The variables were coded in numerical terms, and accordingly, a code sheet was produced. The information was checked for outliers, duplications, redundancy, and missing values and cleaned accordingly. We found missing data of 88 participants which were removed from the final analysis. R- Software v 4.1.0 was used for data analysis. The Chi-square test was applied for ordinal data and a p-value of <0.05 was considered statistically significant for this purpose. Logistic regression analysis was done for the binary outcome variable taking plausible factors of depression. 

## Results

In our study, we included 1500 participants who belonged to classes 9th to 12th. Among those, 88 were discarded due to some missing data. So finally, 1412 participants were included for the analysis purpose. The mean age of the study participants was 16.46 ± 1.22 years. 

Table 1 shows that out of 1412 participants, 814 (57.6%) were boys and 598 (42.4%) were girls. Nearly half of the study participants i.e. 47.2% (672) belong to the age group of 16-17 years and almost equal number of participants are in the age group of 14-15 years (367, 26%) and >17 years (373, 26.4%). 368 participants were studying in class-9th, 326 participants in class-10th, 345 participants in class-11th and 373 participants in class 12th. We also found that the education level majority of the participant's fathers (86.8%, 1225) was above graduation, and about 40.9% (577) of participant’s fathers were engaged in services (private/Government) followed by 31.4%, (444) who were engaged in professional jobs. On the other hand, we reported that 60.6% (856) of participant’s mothers were graduates and above and 11.8% (166) studied only up to middle school. Most of the mothers of the study participants (61.4%, 868) were homemakers and only a few i.e. 1.4% (20) were engaged in business activities. Out of 1412 participants, 910 (64.4%) belonged to nuclear families while 502 (36.6%) belonged to joint families. Nine hundred and forty-six (67%) of participants belonged to lower socio-economic class as compared to merely three (0.2%) participants who belonged to higher socio-economic class. In total, 45.3% (640) of participants had a high screen time. Regarding the activity of students, 44.5% (626) of participants were more active while 40.1% (569) of participants were moderately active, and 15.3% (217) of participants had a sedentary lifestyle. While only 1.7% (24) of participants had experienced failure in their academic performance in the previous year, the majority of the participants (1388, 98.3%) never had a failed experience.

**Table 1:** Distribution of study participants as per socio-demographic profile, academic performance, screen time, and outdoor activity (N=1412)

Characteristic	N	Percentage
Age group		
14-15	367	26%
16-17	672	47.6%
>17	373	26.4%
Gender		
Female	598	42.4%
Male	814	57.6%
Class of Study		
9^th^	368	26%
10^th^	326	23.1%
11^th^	345	24.4%
12^th^	373	26.5%
Outdoor Activity (Playing outdoor games)		
<1 day/week - Sedentary	217	15.4%
1-4 days/week - Moderately active	569	40.1%
≥5 Days days/week - Active	626	44.5%
Result (of previous academic performance)		
Fail	24	1.7%
Pass	1388	98.3%
Father Education		
up to middle	20	1.4%
up to intermediate	167	11.8%
Graduate and above	1225	86.8%
Mother Education		
up to middle	166	11.8%
up to intermediate	390	27.6%
Graduate and above	856	60.6%
Father Occupation		
Professional	444	31.4%
Private/Government Service	577	40.9%
Business	287	20.3%
Others	104	7.4%
Mother occupation		
Professional	185	13.1%
Private/Government Service	201	14.2%
Business	20	1.4%
Others	138	9.8%
Homemaker	868	61.5%
Screen time (Spending time while using electronic gadgets like mobile, tablets, laptops, tv etc.)		
Low (≤ 2 hrs/ day)	772	54.7%
High (> 2 hrs/ day)	640	45.3%
Family Type		
Nuclear	910	64.4%
Joint	502	36.6%
Socio-Economic Status		
Upper Class -I	3	0.2%
Upper middle Class- II	42	3%
Middle Class -III	100	7.1%
Lower middle Class- IV	321	22.7%
Lower Class -V	946	67%

**Figure 1:**
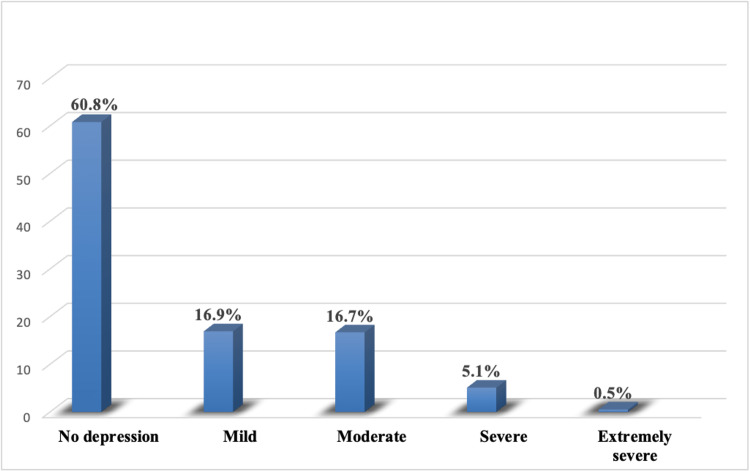
Prevalence of depression among study participants

Figure 1 shows that 239 (16.9%) participants were screened to have mild depression, 236 (16.7%) had moderate depression, 72 (5.1%) had severe depression and 7 (0.5%) had extremely severe depression. The remaining 858 (60.8%) participants did not have any sort of depression.

**Figure 2:**
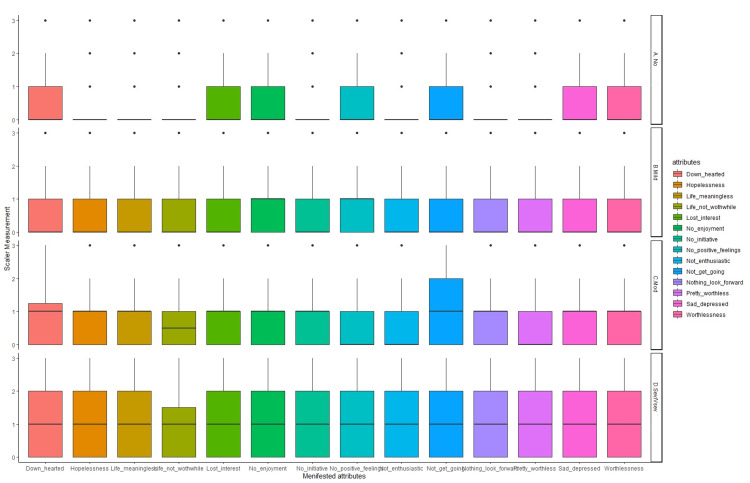
Depression attributes as per depression category (i.e. mild, moderate, severe, and no depression)

Figure 2 depicts the Box-plot distribution of attributes of depression with respect to the levels of depression. It was observed that symptoms were not much pronounced in participants with mild depression while participants with moderate levels of depression lacked interest in initiating any activity. In comparison, participants with severe levels of depression showed all other symptoms equally, with the exception of only a few harboring the feeling of their life not being worthwhile.

Table 2 shows the various factors that influence the occurrence of depression in the study population. We observe that depression was more prevalent among the age group of 16-17 years, as compared to other age groups. It is also more common in study participants living in a nuclear family and belonging to higher socio-economic status. It is almost equally present among the study participants of all classes. However, it was seen that this finding was not significant. It was also observed that the mother’s education was significantly associated with depression.

**Table 2:** Factors influencing the occurrence of depression

Characteristic	Depression present, N = 554	No depression, N = 858	p-value
Age group			0.75
14-15	143 (25.8%)	224 (26.1%)	
16-17	270 (48.7)	402 (46.9%)	
>17	141 (25.5%)	232 (27.03%)	
Gender			0.8
Female	237 (43%)	361 (42%)	
Male	317 (57%)	497 (58%)	
Class of Study			0.7
9^th^	143 (26%)	225 (26%)	
10^th^	126 (23%)	200 (23%)	
11^th^	144 (26%)	201 (23%)	
12^th^	141 (25%)	232 (27%)	
Outdoor Activity			0.6
<1 d/w	79 (14%)	138 (16%)	
1-4 d/w	228 (41%)	341 (40%)	
≥5 Days a week	247 (45%)	379 (44%)	
Result (of previous academic performance)			0.2
Fail	6 (1.1%)	18 (2.1%)	
Pass	548 (99%)	840 (98%)	
Father Education			0.3
up to middle	11 (2.0%)	9 (1.0%)	
up to intermediate	63 (11%)	104 (12%)	
Graduate and above	480 (87%)	745 (87%)	
Mother Education			0.017
up to middle	49 (8.8%)	117 (14%)	
up to intermediate	165 (30%)	225 (26%)	
Graduate and above	340 (61%)	516 (60%)	
Father Occupation			0.3
Professional	173 (31%)	271 (32%)	
Private/Government service	240 (43%)	337 (39%)	
Business	100 (18%)	187 (22%)	
Others	41 (7.4%)	63 (7.3%)	
Mother occupation			0.6
Professional	69 (12.5%)	116 (13.5 %)	
Private/Government service	88 (15.9%)	113 (13.2%)	
Business	7 (1.2%)	13 (1.5%)	
Others	57 (10.3%)	81 (9.4%)	
Homemaker	333 (60.1%)	535 (62.4%)	
Screen time (Spending time while using electronic gadgets like mobile, tablets, laptops, tv etc.)			0.82
Low (≤ 2 hrs/ day)	307 (55.1%)	465 (54.4%)	
High (> 2 hrs/ day)	250 (44.9%)	390 (45.6%)	
Family Type			0.74
Nuclear	360 (65%)	550 (64.1%)	
Joint	194 (35%)	308 (35.9%)	
Socio-Economic Class			0.5
Upper Class- I	1 (0.2%)	2 (0.2%)	
Upper middle class- II	19 (3.4%)	23 (2.6%)	
Middle class - III	40 (7.2%)	60 (7%)	
Lower middle class- IV	113 (20.4%)	208 (24.2%)	
Lower class - V	381 (68.7%)	565 (65.8%)	

Table 3 shows the inter-relationship between various determinants having an effect on depression. It was observed that, by keeping the female gender as the reference category, the odds of males suffering from depression is 0.97 times that of females. Similarly, by keeping the time spent outdoors as a reference category, it was observed that the odds of those engaged in outdoor activities for 1-4 days and more than five days a week suffering from depression was 1.17 times and 1.14 times with that having less than one day a week of outdoor activities. By keeping the participants' father’s graduate and above degrees as the reference category, it was observed that the odds of those whose fathers had studied up to intermediate level and middle school respectively suffering from depression was 0.94 times and 1.90 times than those whose fathers had graduate and above degrees. However, both these findings were statistically insignificant as the p values were found to be 0.717 and 0.158 respectively, which was more than 0.05. Similarly, by keeping those whose fathers had a business, as the reference category, it was observed that the odds of those whose fathers were in other jobs, professional jobs, and Government/private services the depression was 1.22 times, 1.19 times, and 1.33 times respectively than those whose fathers had a business. 

**Table 3:** Interplay of various determinants on causation of depression

Variable		No depression	Depression present	OR (univariable)	OR (multivariable)
Gender	Female	361 (60.4)	237 (39.6)	-	-
	Male	497 (61.1)	317 (38.9)	0.97 (0.78-1.21, p=0.793)	0.97 (0.78-1.20, p=0.757)
Outdoor activity	<1 day/Week	138 (63.6)	79 (36.4)	-	-
	1-4 days/Week	341 (59.9)	228 (40.1)	1.17 (0.85-1.62, p=0.347)	1.18 (0.86-1.64, p=0.315)
	≥5 Days a week	379 (60.5)	247 (39.5)	1.14 (0.83-1.57, p=0.427)	1.14 (0.83-1.58, p=0.410)
Father education	Graduate and Above	745 (60.8)	480 (39.2)	-	-
	Up to intermediate	104 (62.3)	63 (37.7)	0.94 (0.67-1.31, p=0.717)	0.97 (0.68-1.35, p=0.839)
	Up to middle	9 (45.0)	11 (55.0)	1.90 (0.78-4.74, p=0.158)	2.33 (0.93-5.97, p=0.070)
Father occupation	Business	187 (65.2)	100 (34.8)	-	-
	Others	63 (60.6)	41 (39.4)	1.22 (0.76-1.93, p=0.405)	1.30 (0.81-2.08, p=0.272)
	Professional	271 (61.0)	173 (39.0)	1.19 (0.88-1.63, p=0.261)	1.26 (0.92-1.74, p=0.154)
	Private/Government Service	337 (58.4)	240 (41.6)	1.33 (0.99-1.79, p=0.056)	1.42 (1.05-1.93, p=0.024)

## Discussion

Adolescents in the transition phase are a highly vulnerable age group and more prone to suffering from mental health morbidities like depression. Because of this, they need extra care and understanding of this situation. There is also a lack of trained personnel with the required expertise in this field which leads to difficulty as well as delay in diagnosis of the mental health problems like depression. As a result of this, the real quantum of the problem is usually missed. Delay in seeking treatment and care as well as the high cost of medications and treatment makes this situation precariously alarming. The current study’s major focus was to assess the burden of depression among school-going adolescents.

The prevalence of any level of depression (may it be mild depression, moderate depression, severe depression, or very severe depression) in our study participants was found to be around 39%. Studies conducted in some parts of India have found the prevalence among adolescents to be around 40%, which is in line with what we have found in our current study [11,12]. Many studies conducted in various parts of the world have found the prevalence of depression ranges between 10% and 27% [13,14]. Some Indian studies have found this prevalence to be more than what we have found in our study. They have found the prevalence of depression of around 50% [15]. This difference in values found by many researchers may be attributed to different types of scales used which in turn use various kinds of diagnostic criteria (e.g. Diagnostic and Statistical Manual - DSM III, IV, etc). 

We also found in our results that as age increased, the prevalence of depression among our study participants also got increased but this difference was not statistically significant. There are many studies reflecting the adolescent age group being an important determinant for the causation of psychosocial problems in them [16-20].

In our study, we found that as the education level of the father and mother increased, there was an increased prevalence of depression. This difference was statistically significant when we merely compared the mother’s education level with that of the presence or absence of depression. This was in contrast to what Arroyo-Borrell et al. found in their study. They found with increasing maternal education, the prevalence of these mental health problems decreased significantly [21]. In our study, we also found that the participants whose fathers were in professional occupations or were in private or Government service were having higher levels of depression. In India, it is assumed that a child should pursue a career with what their parents say. The more educated the parents are, the more they set highly ambitious goals for their wards to achieve which leads to increased chances of depression. In the case of mothers’ occupation, higher levels of occupation exhibited lower levels of depression.

We found that our study participants belonging to nuclear families exhibited higher levels of depression. In joint families, members have always been protective as well as affectionate toward the other members of the family which also helps to promote mental health well-being [22]. They act as buffers for adolescents against various stressful events in this modern world [23]. Whereas in the nuclear family, there is no such buffer. In this study, we also found a greater prevalence of depression at lower levels of screen time than the higher levels. We also found lower levels of depression among participants who were leading an active lifestyle.

In depression, we found symptoms like “hopelessness”, “life not being meaningful anymore”, “life not being worthwhile”, “difficulty to find the drive to do things”, “not being enthusiastic about anything”, “not having anything to look forward to and life being pretty worthless”, being absent in participants who were found to be not having depression. But these symptoms showed up first in participants suffering from mild depression and its severity increased in participants suffering from severe to very severe depression (as found by our screening tool). So, we found these symptoms to be the earliest markers of depression in our study. As we can observe, these symptoms are majorly emotional and not overtly behavioral. This fact was also reported by Skinner [24]. There were symptoms like lack of self-confidence, downhearted, losing all interest in worldly pleasures, not enjoying the things they were previously enjoying, having no positive feelings, having no interest in doing anything, being depressed, and lastly feeling worthless which we found in our participants, screened of not having depression as well as mild and moderate depression. There was an interesting finding in the sense that the severity of these symptoms increased in severe and very severe forms of depression. These findings were corroborated by the findings of Zimmerman et al. They found the symptoms consistently associated with depression being suicide tendency, low mood, and anhedonia [25]. Understanding these symptoms is important as anyone having these symptoms requires urgent medical care.

We consider the sampling frame and population representation to be substantial. For the sampling units also, all attempts were made to include multiple strata like government and public schools and varied representations of different classes of study. The few limitations that we can think of in this study are to start with as the data collection modality was a self-administered questionnaire, different interpretations of the same questions were possible and thus might have created a cognitive bias among the study participants. Secondly, student data from rural schools were not included and the area of residence could have been a better predictor variable and was missed due to the constraints associated with operational feasibility. Lastly, depression is a complex psychological construct in general and the whole dimension of which might not be captured or quantified with certainty and several other psychometric analyses might completely show the findings.

## Conclusions

Hence, we conclude that the prevalence of depression among school-going adolescents is 39% and mother’s education was the significant determinant for depression among the adolescents. We hereby also recommend that the teachers may be trained about the cues for early identification of this problem in students and a holistic approach should be followed involving parents and teachers with the help of counselors to curb this problem.
